# ETHNIC AND RACIAL DIFFERENCES IN END-OF-LIFE CARE IN TEXAN COLORECTAL CANCER DECEDENTS

**DOI:** 10.1093/geroni/igae098.3840

**Published:** 2024-12-31

**Authors:** Bethany Leach, Efstathia Polychronopoulou, Yong-Fang Kuo, Mukaila Raji, Myrna Serna

**Affiliations:** University of Texas Medical Branch at Galveston, Galveston, Texas, United States; The University of Texas Medical Branch, Galveston, Texas, United States; University of Texas Medical Branch, Galveston, Texas, United States; University of Texas Medical Branch, Galveston, Galveston, Texas, United States; The University of Texas Medical Branch, Galveston, Texas, United States

## Abstract

There are known racial and ethnic disparities in advance care planning (ACP) and downstream outcomes. We hypothesized that among Texan Medicare decedents with colorectal cancer (CRC), there would be racial and ethnic disparities in ACP, palliative care (PC) consultation and hospice enrollment. Data from the Texas Cancer Registry linked with Medicare claims records for patients age ≥66 years who died 6 months to 5 years after a primary CRC diagnosis between 2010 and 2018 was analyzed. Number (proportion) of Non-Hispanic Black (NHB), Non-Hispanic White (NHW) and Hispanic decedents with ACP, PC consultation and hospice enrollment in the last 30 days of life are reported and chi-square tests used to assess statistically significant differences. A multivariable logistic regression model was used to assess racial/ethnic differences in odds of hospice enrollment, adjusting for CRC stage and other demographic variables. Among 5,757 Medicare decedents, ≤2.2% had ACP with no significant racial/ethnic differences noted. While NHBs were most likely to receive PC consultation (106 (18%), p=0.017), they were least likely to be hospice enrolled (369 (62%), p< 0.001). The multivariable logistic regression model showed NHBs had decreased odds of hospice enrollment when compared to NHWs (OR 0.67, 95% CI 0.55-0.81). Among Medicare decedents with CRC, there are low rates of ACP and racial/ethnic disparities in PC consultation and hospice care. More and earlier ACP is needed to provide patient-centered care. While more NHBs received PC consultation in the last 30 days of life, it is unclear if PC consultations were provided earlier in life course.

